# A Prospective Study of Specialized Coagulation Parameters in Admitted COVID-19 Patients and Their Correlation With Acute Respiratory Distress Syndrome and Outcome

**DOI:** 10.7759/cureus.17463

**Published:** 2021-08-26

**Authors:** Tushar Sehgal, Nitesh Gupta, Santvana Kohli, Aditi Khurana, Jasmita Dass, Sahil Diwan, Mahendran A J, Maroof Khan, Mukul Aggarwal, Arulselvi Subramanian

**Affiliations:** 1 Laboratory Medicine, All India Institute of Medical Sciences, New Delhi, IND; 2 Internal Medicine • Pulmonology, Vardhman Mahavir Medical College, Safdarjung Hospital, New Delhi, IND; 3 Anesthesiology, Vardhaman Mahavir Medical College, Safdarjang Hospital, New Delhi, IND; 4 Emergency Department, Vardhaman Mahavir Medical College, Safdarjang Hospital, New Delhi, IND; 5 Hematology, All India Institute of Medical Sciences, New Delhi, IND; 6 Anaesthesiology, Vardhaman Mahavir Medical College, Safdarjang Hospital, New Delhi, IND; 7 Internal Medicine • Pulmonology, Safdarjung Hospital, New Delhi, IND; 8 Biostatistics, All India Institute of Medical Sciences, New Delhi, IND; 9 Pediatrics • Hematology, All India Institute of Medical Sciences, New Delhi, IND

**Keywords:** covid-19, coagulation abnormalities, sofa score, ards, outcome

## Abstract

Background

Acute respiratory distress syndrome (ARDS) is a frequent complication of COVID-19 and is associated with a component of thrombo-inflammation and cytokine storm. COVID-19 also affects the hemostatic system causing multiple coagulation abnormalities that is a cause of concern and needs to be addressed.

Objective

We aimed to assess coagulation parameters of COVID-19 patients and identify whether they could be used as potential prognostic biomarkers to predict ARDS and immediate outcomes.

Methods

This was a prospective study done on 68 patients at four serial time points. Patients between 18-85 years admitted to the hospital as in-patients and ICU with a confirmed diagnosis of COVID-19 by RT-PCR were included. Exclusion criteria included pregnancy, patients below and above the mentioned age, previously known coagulopathy, systemic anticoagulants or anti-platelet therapy or vitamin K antagonists and moribund patients. Patients were divided into three categories based on SOFA score at admission, presence (group 1) or absence (group 2) of ARDS and outcome (dead or alive). Routine and specialized coagulation tests were performed on patients' platelet-poor plasma at the time of study inclusion (day 0), days 3, 7 and at discharge on STAR Max®3 (Diagnostica Stago France) automated coagulation analyzer and included prothrombin time (PT), international normalized ratio (INR) (STA® -NeoPTimal), activated partial thromboplastin time (APTT) (STA® -Cephascreen), fibrinogen (STA® Liquid Fib), D-dimer (STA® LiatestD- Dimer), Protein C (STA Stachrom® Protein C), Protein S (STA® Latest Free Protein S) and Antithrombin (STA® Chrom ATIII). ELISA did testing for tissue plasminogen activator (Asserachrom® tPA) as per the manufacturer's protocol.

Results

Sixty-eight patients, including 43 (63%) males and 25 (37%) females, with a median age of 48 years (IQR 20-85), were recruited in this study. The incidence of ARDS was 34%, with a mortality of 13%. History of contact with a COVID-19 case was present in 71% (48/68) of the patients. Fever was the most common presenting symptom in 84% (57/68) of the patients. The most common comorbidities were hypertension and diabetes mellitus (DM) in 22% (15/68) and 21% (14/68) of the patients. DM (p=0.07) and chronic obstructive pulmonary disease (COPD) (p=0.03) were significantly associated with ARDS. DM (p=0.02), hypertension (p=0.01), and COPD (p=0.02) were also significantly associated with mortality. APTT was markedly prolonged among non-survivors at day 0 (D0) and D7 (p=0.03, p=0.02). D-Dimer was elevated in 38/68 (56%) patients at D0. D-Dimer levels were significantly higher in non-survivors (p<0.001), in ARDS patients (p=0.001) and patients with higher SOFA scores (p=0.001). ROC curve showed that D-dimer cut-off > 2.13 (AUC of 0.86) and >0.85 (AUC of 0.74) predicts mortality and ARDS, respectively. Among the natural anticoagulants, protein C was significantly associated with a high SOFA score at D0 and D3 (p=0.04).

Conclusion

Diabetes mellitus, hypertension and COPD were associated with poor outcomes. D-dimer levels must be monitored in COVID patients due to their association with ARDS and mortality. We observed that the levels of natural anticoagulants fell during the illness, making them prone to coagulopathies; however, none were seen in this study. Elevated tPA levels were also found in our patients; fibrinolytic therapy may benefit COVID-19 patients suffering from ARDS.

## Introduction

Severe acute respiratory syndrome coronavirus 2 (SARS-CoV-2), the causative agent of coronavirus disease 2019 (COVID-19), was first identified in Wuhan, China, in late December 2019 [1]. This virus belongs to the β-Coronavirus family and is partially like the SARS-CoV and MERS-CoV coronaviruses, which have caused previous epidemics in China and the Middle East, respectively [2]. Since then, COVID-19 has spread throughout the world. The World Health Organization (WHO) first declared a public health emergency of international concern on 30 January 2020 and then formally declared it a pandemic on 11 March 2020 [3]. According to the WHO, it has affected over 196.55 million people worldwide, with more than 4.2 million deaths. The United States has the highest number of reported infections and deaths globally, followed by India, Brazil, Russia, France, and the United Kingdom [4]. The classic symptoms include fever, cough, dyspnea, and loss of taste/smell, albeit some patients may be asymptomatic. Severe disease complications include multi-organ failure, acute respiratory distress syndrome (ARDS), septic shock, and venous thromboembolism [5-8]. ARDS is common amongst patients needing hospitalization, which comprise around 20% of all infected patients [5]. The exact mechanism of pulmonary complications and ARDS of COVID-19 has not been elucidated, but there is a clear component of thrombo-inflammation and cytokine storm [9,10].

There are increasing reports of venous thromboembolism in COVID-19 patients, and arterial thrombosis, including stroke and myocardial infarction, has been described [11-14]. The underlying mechanisms of COVID-19-related coagulopathy are complex. They involve invasion of the vascular endothelial cells via angiotensin-converting enzyme 2 receptor causing endothelialitis, recruitment of inflammatory cells like polymorphs and monocytes with activation of complement and thrombin generation [15]. More than 33% of critical COVID-19 patients' are reported with coagulation abnormalities, including elevated levels of D-dimer [13]. The development of these unusual clots causing coagulation abnormalities and thrombosis is the real concern and needs to be addressed. Our study aimed to perform a comprehensive evaluation of coagulation parameters of patients with COVID-19 pneumonia admitted to our hospital and identify potential prognostic biomarkers of this new disease.

## Materials and methods

Study design

We performed a prospective multicentric study in the ward and ICU of two hospitals, Vardhaman Mahavir Medical College (VMMC) and Safdarjung Hospital, New Delhi, India and All India Institute of Medical Sciences New Delhi, India. The Institute Ethics Committee approved the study. Eighty patients between 18-85 years admitted to the in-patient department (IPD) and ICU with a confirmed diagnosis of COVID-19 was included in this study. The severity of COVID-19 was graded as per the Ministry of Health and Family Welfare (MOHFW), India guidelines [16]. We followed uniform protocol management for all admitted patients [17]. Participants were recruited over two months and represented the majority of patients infected by COVID-19. Laboratory confirmation of SARS-CoV-2 infection was based on a positive reverse-transcriptase-polymerase chain-reaction (RT-PCR) assay. Exclusion criteria included pregnancy, patients below 18 years and above 85 years of age, previously known coagulopathy, systemic anticoagulants or anti-platelet therapy or vitamin K antagonists, moribund patients and patients from whom we did not get consent for participation. Patients were divided into three categories based on sequential organ failure assessment score (SOFA) at admission, presence or absence of ARDS and outcome (alive or dead). Based on the median total maximum SOFA score, patients were divided into group SOFA 0 and group SOFA ≥ 1. The clinical, radiological and cardiac criteria's for defining ARDS are as follows; firstly, the onset of chest symptoms within one week of a known clinical insult or new/worsening respiratory symptoms; secondly, chest imaging showing bilateral opacities that are not fully explained by effusions, lobar/lung collapse, or nodules; and lastly respiratory failure not fully explained by cardiac failure or fluid overload [16]. Group 1 included COVID 19 patients with ARDS (PaO2/FiO2≤300mm Hg) at presentation, and Group 2 included COVID 19 patients with no ARDS (PaO2/FiO2>300 mm Hg) during disease.

Laboratory testing of coagulation parameters

All precautions for handling, transport, packaging, and opening, processing and discarding potentially hazardous samples were followed [18]. We collected blood from a peripheral vein into citrated vacutainer. A blood sample was centrifuged for 15 min at 1500 × g to obtain platelet-poor plasma (PPP). Laboratory tests were performed on PPP at the time of study inclusion (day 0), days 3, 7 and at discharge on STA R Max®3(Diagnostica Stago France) and included prothrombin time (PT), international normalized ratio (INR) (STA® -NeoPTimal) , activated partial thromboplastin time (APTT) (STA® -Cephascreen), fibrinogen (STA® Liquid Fib), D-dimer (STA® LiatestD- Dimer), Protein C (STA Stachrom® Protein C), Protein S (STA® Latest Free Protein S) and Antithrombin (STA® Chrom ATIII). Testing for Tissue plasminogen activator (Asserachrom® tPA) was done by ELISA method. The PPP was aliquoted in a microcentrifuge tube and frozen at -80◦C. Before testing, the sample was thawed at 37C, and ELISA testing was done as per the manufacturer's protocol.

Data collection

All study data were retrieved from an electronic structured case report form where trained case managers prospectively entered patients data. Data including clinical variables, demographics, comorbidities, SOFA score at ICU admission, treatment measures (i.e. corticosteroids, anticoagulation, interleukin-6 receptor antagonist, anti-viral therapy, broad-spectrum antibiotics), supportive therapy (mechanical ventilation, non-invasive mechanical ventilation), length of stay (LOS) in ICU and hospital and ICU mortality were collected. Blood component transfusion [platelet concentrate, fresh frozen plasma and cryoprecipitate] and hemostatic agents (fibrinogen concentrate, prothrombin complex concentrate and tranexamic acid) were also collected. The presence of thrombotic or hemorrhagic events during ward and ICU stay were also recorded.

Statistical analysis

Data were analyzed using Stata 11.2 (Stata Corp 4905 College Station, Texas 47845 USA) and presented as mean±SD, median (range) or frequency (percentage). The adolescents with and without ARDS were compared using the chi-square test or Fisher's exact test for categorical variables, one-way ANOVA or independent t-test for continuous variables following normal distribution, and Kruskal-Wallis or Wilcoxon rank-sum test for parameters following non-normal distribution. Receiver operating characteristic (ROC) curves were generated to identify the cut-offs for coagulation tests for association with ARDS. Univariate and stepwise multivariate logistic regression was used to calculate unadjusted and adjusted odds ratios after assessing multicollinearity and mediators among the variables. A p-value of <0.05 was considered statistically significant.

## Results

Baseline parameters

A total of 80 patients were recruited randomly in this study. However, 68 patients, including 43 (63%) males and 25 (37%) females, were analyzed at four sequential time points. The median age was 48 years (IQR 20-85). History of contact with a COVID-19 case was present in 71% (48/68) of the patients. We classified the patients based on SOFA score at admission, presence or absence of ARDS at presentation and outcome. The severity of COVID-19 was mild in 45 (66%) and severe in 23 (34%) patients. The descriptive statistics of the cohort are presented in table 1 and table 2. The most common comorbidities were hypertension and diabetes mellitus (DM) found in 22% (15/68) and 21% (14/68)of the patients, respectively. Chronic obstructive pulmonary disease (COPD) was present in 4% (3/68) of the patients. Other comorbidities included coronary artery disease and thyroid disorder in 10% (7/68) and 7% (5/68) of the patients, respectively. Seizure disorder, chronic kidney disease, tuberculosis, asthma were present in 3% (2/68) of the patients, while depressive disorder was present in one patient. Fever was the most common presenting symptom found in 84% (57/68) of the patients. Cough, dyspnea, headache/myalgia and running nose were present in 59% (40/68), 41% (28/68), 21% (14/68) and 12% (8/68) of the patients, respectively. Other less common symptoms were sore throat, diarrhoea and loss of smell present in 9% (6/68), 6% (4/68), and 3% (2/68) of the patients, respectively.

**Table 1:** Basic characteristic of study participants concerning SOFA score and ARDS *denotes significant p-value ARDS- Acute respiratory distress syndrome; COPD- Chronic obstructive pulmonary disease; SOFA- Sequential organ failure assessment

Characteristics	Total patients N (%)	SOFA score		p-value	ARDS N=23 (%)	Non-ARDS N=45 (%)	p-value
		0 N=36 (%)	≥1 N=32(%)				
Demographics							
Age (years) ≤48	33/68 (49)	22 (61)	11(34)	0.028*	8 (35)	25 (56)	0.10
>48	35/68 (51)	14 (39)	21(66)		15 (65)	20 (44)	
Gender, Males	43/68 (63)	23 (64)	20 (62)	0.9	15 (65)	28 (62)	0.80
Females	25/68 (29)	13 (36)	12 (38)		8 (35)	17 (38)	
Symptoms							
Fever	57/68 (84)	27 (75)	30 (94)	0.04*	23 (100)	34 (76)	0.01*
Cough	40/68 (59)	17 (47)	23 (72)	0.03*	17 (74)	23 (51)	0.07
Dyspnoea	28/68 (41)	7 (19)	21 (66)	<0.001*	19 (83)	9 (20)	<0.001*
Headache/myalgia	14/68 (21)	2(6)	12 (37)	0.002*	11 (48)	3 (7)	<0.001*
Running nose	8/68 (12)	2 (6)	6 (19)	0.13	6 (26)	2 (4)	0.01*
Sore throat	6/68 (9)	4 (11)	2 (6)	0.67	1 (4)	5 (11)	0.65
Diarrhoea	4/68 (6)	4 (11)	0	0.11	0	4 (9)	0.29
Anosmia	2/68 (3)	2 (6)	0	0.49	0	2 (4)	0.54
Comorbidities							
Hypertension	15/68 (22)	6(17)	9 (28)	0.2	7 (30)	8 (18)	0.23
Diabetes Mellitus	14/68 (21)	4 (11)	10 (31)	0.06	9 (39)	5 (11)	0.007*
Coronary artery disease	7/68 (10)	4(11)	3 (9)	1.0	3 (13)	4 (9)	0.68
COPD	3/68 (4)	0	3 (9)	0.09	3 (13)	0	0.03*
Hypothyroidism	2/68 (3)	2(6)	2 (6)	1.0	2 (9)	2 (4)	0.48
Asthma	2/68 (3)	0	2 (6)	0.21	1(4)	1 (2)	1.0
Seizure disorder	2/68 (3)	2(6)	0	0.49	0	1 (2)	1.0
Chronic kidney disease	2/68 (3)	0	2 (6)	0.21	1(4)	1 (2)	1.0
Tuberculosis	2/68 (3)	1(3)	1(3)	1.0	1(4)	1 (2)	1.0
Hyperthyroidism	1/68 (1)	0	1(3)	0.47	1(4)	0	0.33
Depression	1/68 (1)	1 (3)	0	1.0	0	1(2)	1.0

**Table 2:** Basic characteristics of study participants with respect to outcome *denotes significant p-value COPD- Chronic obstructive pulmonary disease

Characteristics	Total patients N (%)	Outcome		p-value
		Alive , N=59 (%)	Died, N=9 (%)	
Demographics				
Age (years) ≤48	33/68 (49)	30 (51)	3 (33)	0.47
>48	35/68 (51)	29 (49)	6 (67)	
Gender, Males	43/68 (63)	36 (61)	7 (78)	0.46
Females	25/68 (29)	23 (39)	2 (22)	
Symptoms				
Fever	57/68 (84)	48 (81)	9 (100)	0.33
Cough	40/68 (59)	34 (58)	6 (67)	0.72
Dyspnoea	28/68 (41)	21 (36)	7 (78)	0.02*
Headache/myalgia	14/68 (21)	11 (19)	3 (33)	0.37
Running nose	8/68 (12)	5 (8)	3 (33)	0.06
Sore throat	6/68 (9)	5 (8)	1 (11)	1.0
Diarrhoea	4/68 (6)	4 (7)	0	1.0
Anosmia	2/68 (3)	2 (3)	0	1.0
Comorbidities				
Hypertension	15/68 (22)	10 (17)	5 (56)	0.02*
Diabetes Mellitus	14/68 (21)	9 (15)	5 (56)	0.01*
Coronary artery disease	7/68 (10)	5 (8)	2 (22)	0.2
COPD	3/68 (4)	0	3 (33)	0.002*
Hypothyroidism	2/68 (3)	4 (7)	0	1.0
Asthma	2/68 (3)	2 (3)	0	1.0
Seizure disorder	2/68 (3)	2 (3)	0	1.0
Chronic kidney disease	2/68 (3)	1 (2)	1 (11)	0.24
Tuberculosis	2/68 (3)	1 (2)	1 (11)	0.24
Hyperthyroidism	1/68 (1)	1 (2)	0	1.0
Depression	1/68 (1)	1(2)	0	1.0

Coagulation parameters

**Figure 1:**
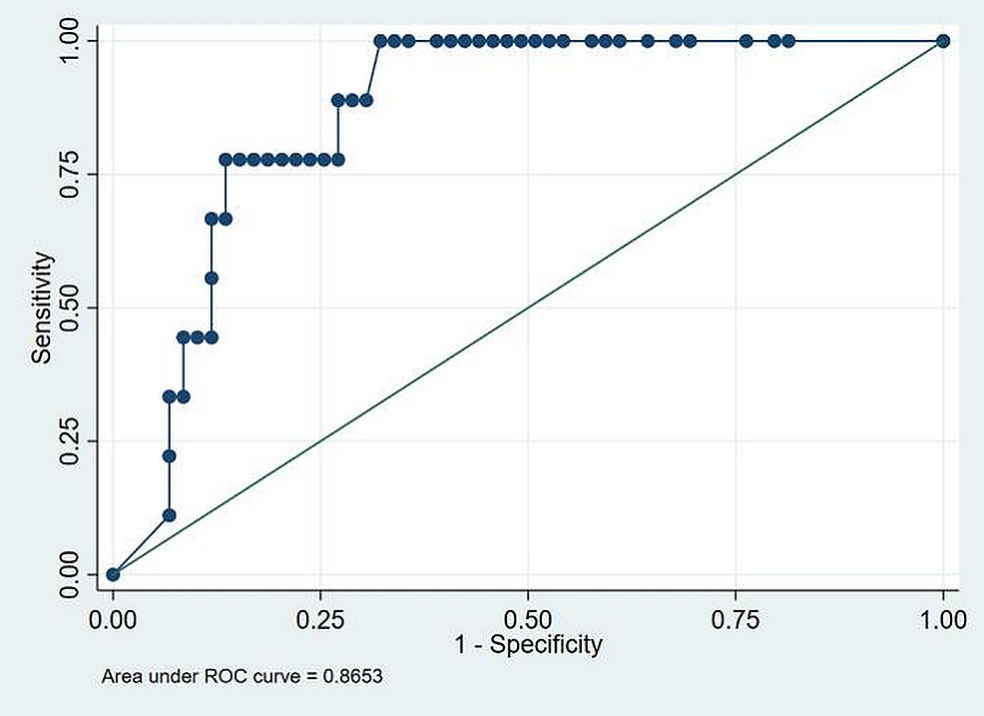
ROC to identify the cut-off for D-dimer to predict risk for mortality. D-dimer had the highest discriminating ability with AUC of 0.86, and the cut-off being 2.13 with sensitivity of 78.2% and specificity of 76%. ROC-receiver operating characteristic curve; AUC-area under curve

**Figure 2:**
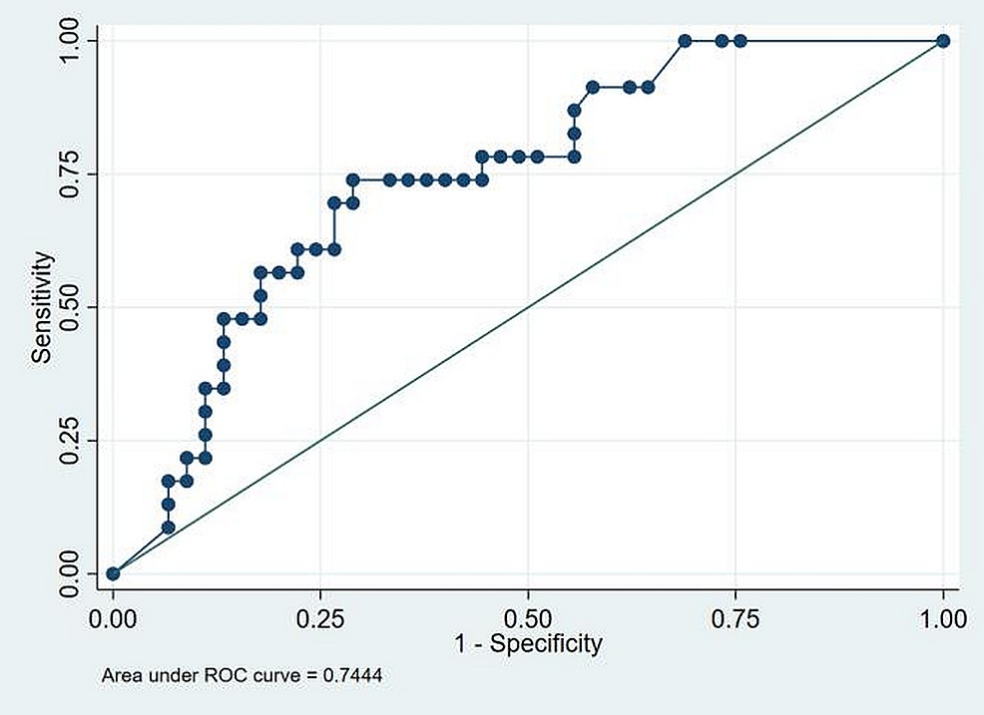
ROC to identify the cut-off for D-dimer to predict risk for ARDS. D-dimer had the highest discriminating ability with AUC of 0.74, and the cut-off being 0.85 with sensitivity of 73.9% and specificity of 71.1%. ROC-receiver operating characteristic curve; AUC-area under curve

The descriptive coagulation and fibrinolytic parameters of the cohort are presented in table 3. The results obtained were analyzed with SOFA score, outcome (alive/dead) and ARDS. Mean PT (seconds) among non-survivors at D0, D3 and D7 were 13.8 ± 1.72, 15.4 ± 2.33 and 15.6 ±3.6. The values were higher in non-survivors than in survivors at D3 and D7. However, the result was not statistically significant (p=0.37, p=0.23 respectively). Median APTT values (seconds) among non-survivors at D0, D3 and D7 were 30.7 (26.2-40.6), 29.6 (24.9-35.8) and 33.7 (27.2-41.6). The values were higher in non-survivors than in survivors, p=0.03, p=0.14, p=0.02, respectively. Median fibrinogen levels (mg/dl) among patients with high SOFA score at D0, D3 and D7 were 286.5 (37.6-697), 236 (28.7-588) and 223.5 (40-467). The values were increased than in patients with low SOFA scores, p=0.15, p=0.46, p=0.10, respectively. Median D-dimer values (ng/ml) in non-survivors were 6.3 (1-20), 7.6 (0.93-20) and 9.85 (1.34-20) at D0, D3 and D7 respectively. The values were higher than in survivors, respectively, p<0.001, p<0.001, p<0.001. Median D-dimer values (ng/ml) in patients with high SOFA score at D0, D3 and D7 were 1.98 (0.27-20), 2.21 (0.27-20) and 2.43 (0.3-20). The values were higher than in patients with low SOFA scores, p<0.001, p=0.001, p=0.004, respectively. Media D-dimer values (ng/ml) in ARDS group were 2.35 (0.3-20), 3.5 (0.27-20) and 3.6 (0.3-20). The values were higher than in the non-ARDS group, p=0.001, p<0.001, p=0.002, respectively. Mean Protein C values (%) among patients with high SOFA scores at D0, D3 and D7 were 64.94±24.8, 62.2 ±30.7 and 60.4±25.7, respectively. These values were lower than in patients with low SOFA scores, p=0.04, p=0.04, p=0.07. ROC comparison concerning mortality with D-dimer levels upon admission is shown in figure 1. Using the cut-off value of >2.13, we found that D-dimer levels upon admission for in-hospital mortality have an area under the curve (AUC) of 0.86. The sensitivity and specificity are 78.2% and 76%, respectively. Figure 2 shows ROC for D-Dimer with ARDS as the outcome. The cut-off is 0.85 with a sensitivity of 73.9% and specificity of 71.1% with an AUC of 0.74.

**Table 3:** Results of Coagulation and Fibrinolytic parameters Normal values-PT (prothrombin time): 11-14 sec; APTT (activated partial thromboplastin time): 24-35 seconds; Fibrinogen: 200-400 mg/dl; D-Dimer: ≤0.5 µg/ml; Protein C:70-130%; Free Protein S:60-140%, Antithrombin:80-120%; tPA (Tissue Plasminogen Activator) 2-12 ng/ml; INR- International normalized ratio *shows significant p-value

Parameters	Outcome		p-value	SOFA score		p-value	ARDS	Non-ARDS	p-value
	Survivor	Non-survivor		0	≥1				
PT D0	13.9 ± 7.7	13.8 ± 1.72	0.96	14.58±9.81	13.1±1.4	0.41	13.45±1.45	,14.14±8.8	0.71
D3	13.4 ± 5.99	15.4 ± 2.33	0.37	14.09±7.57	13.25±2.0	0.55	13.59±2.25	13.76±6.8	0.91
D7	13.7±4.4	15.6 ±3.6	0.23	13.8 ±5.79	14.1±2.48	0.81	14.42±2.79	13.69±5.1	0.54
Discharge	13.3±2.77	0	-	14.1± 3.99	12.7± 1.04	0.94	13.1± 1.04	13.44± 3.16	0.49
INR D0	0.93 ± 0.2	1.06 ± 0.17	0.09	0.92±0.25	0.98±0.12	0.23	1.01±0.13	0.92±0.22	0.1
D3	1 ± 0.46	1.1 ± 0.17	0.39	1.04±0.59	0.98±0.15	0.54	1±0.17	1±0.53	0.89
D7	1±0.34	1.05±0.39	0.75	1.02±0.45	1.01±0.22	0.96	1±0.25	1±0.39	0.83
Discharge	0.99±0.21	0	-	1.04± 0.3	0.94± 0.08	1.0	0.97±0.07	0.99±0.24	0.44
APTT D0	27.5 (20,120)	30.7 (26.2,40.6)	0.03*	27.4(21.8,120)	28.4 (20,120)	0.53	29 (20,120)	27.5 (20,120)	0.15
D3	26.7 (20,120)	29.6 (24.9,35.8)	0.14	27.3 (20,120)	25.9 (20,41.8)	0.10	25.9 (20,41.2)	27.4 (20,120)	0.17
D7	28.5 (20,61.6)	33.7 (27.2,41.6)	0.02*	29.1 (20,61.6)	28.5 (20,41.6)	0.83	28.4 (20,41.6)	28.9 (20,61.6)	0.76
Discharge	25.7(20,46)	0	-	25.7 (22.9,46)	25.7 (20± 29.6)	0.56	27.2 (24.4,29.6)	25.3 (20,46)	0.61
D-Dimer D0	0.5 (0.27,20)	6.3 (1,20)	<0.001*	0.4 (0.27,20)	1.98 (0.27,20)	<0.001*	2.35 (0.3,20)	0.46 (0.27,20)	0.001*
D3	0.54 (.27,20)	7.6 (0.93,20)	<0.001*	0.42 (0.27,20)	2.21 (0.27,20)	0.001*	3.52 (0.27,20)	0.53 (0.27,20)	<0.001*
D7	0.69 (0.2,20)	9.85 (1.34,20)	<0.001*	0.54 (0.3,20)	2.43 (0.3,20)	0.004*	3.6 (0.3,20)	0.55 (0.27,20)	0.002*
Discharge	0.35 (0.27,20)	0	-	0.34(.27,20)	0.35(0.27,9.39)	0.82	0.64 (0.27,1.36)	0.31 (0.27,20)	0.53
Fibrinogen D0	260 (29,704)	231.5 (37.6,484)	0.47	235.5 (29,704)	286.5 (37.6,697)	0.15	240 (37.6,697)	260 (29,704)	0.81
D3	210 (28.7,701)	276 (142,572)	0.20	210 (32.4,701)	236 (28.7,588)	0.46	232 (28.7,588)	211.5 (32.4,701)	0.74
D7	219 (22.9,389)	219.5 (87,467)	0.49	187 (22.9,370)	223.5 (40,467)	0.10	220 (40,467)	205.5 (22.9,389)	0.50
Discharge	192 (133,479)	0	-	200.5 (137,433)	171 (133,479)	0.88	161 (159,171)	222.5 (133,479)	0.31
Protein C D0	78.6±33.25	65.8±27.9	0.50	81±37.8	64.94±24.8	0.04*	82.9±39.8	77.2±26.8	0.05
D3	73.6±36.8	65.1±19.6	0.79	77.7±37.9	62.2±30.7	0.04*	80.19±38.99	72±31.4	0.05
D7	73.4±29.6	52.1 ±35.7	0.07	77.3±34.2	60.4±25.7	0.07	66.3±27.17	66.6±36.5	0.23
Discharge	63.15±18	0	-	68.3±16.88	58.7± 19.05	0.28	48.3±8.0	67.6±17.99	0.09
Protein S D0	64.3±20.8	58.8±9.86	0.53	62±20.4	67.6±18.2	0.21	69.7±18.2	63.69±20.5	0.43
D3	60.3±26.3s	55.6±14.02	0.67	60.45±25.39	67.5±21.88	0.05	66.6±23.6	61.3±23.7	0.05
D7	59.3±25.3	53±20.17	0.74	54.88±27.1	65.1±20.6	0.22	66.2±21.47	54.7±25.7	0.16
Discharge	69±15.89	0	-	69.5± 12.02	68.5± 24.74	-	51±0	75±12.76	-
Antithrombin D0	82.2±26.5	67.5±25.9	0.14	79.9±28.8	80.8±24.7	0.89	79.04±25.74	81.09±27.4	0.77
D3	76.21±25.2	66.37±17.22	0.53	76.8±24.7	73.8±24.0	0.64	72.0±22.3	77.2±25.3	0.43
D7	53.83±23.22	60.71±23.83	0.06	75.3±26.72	66.2±20.17	0.16	63.8±21.5	74.5±24.5	0.12
Discharge	71.46±27.37	0	-	80.1±28.9	64±25.7	0.25	44±5.2	69.7±25.8	0.01*
tPA D0	18.2±11.53	17.6±15.9	0.90	16.77±12.14	19.64±12.03	0.34	19.6±11.9	17.3±12.2	0.47
D3	16.95 ±11.3	14.74±13.5	0.61	15.3±11.18	18.27±12	0.30	17.9±12.08	16.07±11.4	0.53
D7	16.27±12.12	23.98±16.6	0.12	16.48±12.8	18.35±13.3	0.60	17.7±12.8	17.2±13.3	0.90
Discharge	13.71±11.56	0	-	10.4±5.76	16±14.4	0.80	21.73±19.3	11.0±7.6	0.40

Treatment and outcomes

The treatment of COVID-19 patients consists of antipyretics, oxygen therapy, steroids, low molecular weight heparin, anti-viral therapy (remdesivir), IL-6 antagonist (Tocilizumab) and broad-spectrum antibiotics. The median length of stay for all patients was 10 days (IQR, 1-30). The mean length of stay was higher (12.8±6.8 days) in patients with high SOFA scores (p=0.002). Similarly, in patients with ARDS at presentation, the mean length of stay was higher (13.4±7.6 days) (p=0.003). There were nine deaths overall; all were in the ARDS group, while none were in the non-ARDS group (p=0.001). In the ARDS group, 8 (35%) patients were given oxygen therapy, while in the non-ARDS group, 4 (9%) required oxygen therapy. Non-invasive ventilation was required by 6 (26%) in the ARDS group and one patient (2%) in the non-ARDS group. 9 (39%) patients in the ARDS group needed intermittent positive pressure ventilation (IPPV). All 9 of them (100%) eventually succumbed to the disease (p=<0.001). No patient in the non-ARDS required IPPV. Also, no mortality was recorded in this group of patients. No patient in this study had an episode of thrombosis or bleeding. The Association of various parameters with ARDS was evaluated by univariate followed by multiple logistic regression analysis. Headache/myalgia and dyspnea emerged as significant independent variables associated with ARDS (Table 4).

**Table 4:** Association of various parameters with ARDS by logistic regression analysis OR: odds ratio; CI: confidence interval; ARDS- Acute respiratory distress syndrome; PT- Prothrombin time *shows the significant p-value

Variables	ARDS N=23 (%)	Non- ARDS N=45 (%)	Unadjusted OR (95% CI)	p-value	Adjusted OR (95% CI)
Cough	17 (74)	23 (51)	2.71(0.90-8.1)	0.07	-
Headache	11 (48)	3 (7)	12.83(3.07- 53.55)	<0.001	7.38 (1.39-39.09)
Dyspnoea	19 (83)	9 (20)	19 (5.16-69.8)	<0.001	13.9(3.53-54.90)
Diabetes	9 (39)	5 (11)	5.14(1.47-17.97)	0.01	-
Hypertension	7 (30)	8 (18)	2.02 (0.62-6.53)	0.23	-
PT (Day 0)	23 (100)	45 (100)	1.66(0.59-4.61)	0.33	-
D-Dimer (Day 0)	23 (100)	45 (100)	0.166 (0.054-0.51)	0.002	-

Comparison of characteristics among those who died and survived 

There were overall 9 (13%) deaths; all of them had severe COVID-19 infection with ARDS. Those who died compared to those who survived were older (p=0.47), more males than females (p=0.46), had hypertension (p=0.02) and DM (p=0.01) and COPD (p=0.002). Among the non-survivors, higher values of both APTT and D-Dimer were noted. Both were statistically significant at D0, D3 (D-dimer only) and D7 (Table 3). Moreover, among non-survivors higher values of PT, INR, and tPA and lower values of natural anticoagulants Protein C, Protein S and Antithrombin were noted. However, the results were not statistically significant at D0, D3 and D7 (Table 3).

## Discussion

This study comprehensively evaluated the clinical characteristics and coagulation parameters of all confirmed COVID-19 patients regarding SOFA score, ARDS, and mortality. The life-threatening form of respiratory failure, ARDS, is a frequent complication in COVID-19 [12]. The severity of ARDS is classified into mild, moderate, and severe categories, depending on the degree of hypoxemia [19]. Patients with moderate-to-severe ARDS require invasive mechanical ventilation (IVM) and have a poor prognosis. The incidence of ARDS in COVID-19 patients ranges from 2-68%, with a mortality rate of 0-28% [20]. The incidence of ARDS in this study was 34%, with a mortality rate of 13%. Autopsy data in COVID-19 patients suggest exudative diffuse alveolar damage with proteinaceous alveolar exudates and edema, vascular congestion, and focal fibrin deposition with pneumocyte hyperplasia. These findings suggest that pulmonary coagulopathy starts early in the disease itself. SARS-CoV2 infects the type II pneumocytes via angiotensin-converting enzyme 2 (ACE2) receptors. Due to the widespread presence of ACE-2 receptors in the lung, there is damage to the large surface area of the alveoli, leading to hypoxemia and extensive vascular damage due to the juxtaposition of type II pneumocytes to the vessels. The infection also causes a massive cytokine storm. There is an increased expression of tissue factors on endothelium and inflammatory cells, including neutrophils and macrophages. These phenomena ultimately lead to intrapulmonary activation of the coagulation cascade [21].

We found that higher patient age was significantly associated with SOFA score (p=0.02). Moreover, ARDS and mortality were also more common in patients with higher age. However, it was not statistically significant (p=0.10, p=0.47 respectively). Wu et al. also showed that older age was associated with a greater risk of developing ARDS and death. This may be explained by the fact that older age is associated with a decline in immune competence, and therefore, less robust immune responses are produced [12]. In our patients, the role of gender in higher SOFA scores, development of ARDS and clinical outcomes was observed. Males had higher SOFA scores, ARDS and higher mortality than females; however, the results were not statistically significant. All patients who died were ICU patients with severe COVID-19 disease. Jin JM et al. also showed gender is a risk factor for higher severity and mortality in patients with COVID-19, independent of age and susceptibility [22]. In this study, the most common presenting symptoms (in that order) were fever, dry cough, dyspnea, and headache/myalgia found in 84%, 59%, 41% and 21% of the patients. More than 50% of the patients who presented with fever, dry cough, and dyspnea had high SOFA scores (p=0.04, p=0.03, p<0.001, respectively), while 37% of the patients with headache and myalgias had high SOFA scores (p=0.002). Fever, dyspnea and headache/myalgias were significantly associated with ARDS (p=0.01, p<0.001, and p<0.001, respectively). Only dyspnea was associated with both ARDS (p<0.001) and mortality (p=0.02). Other less common symptoms were running nose, sore throat, diarrhoea, and loss of smell in 12%, 9%, 6%, and 3% of the patients. Wu et al. in their study on 201 patients, showed that the most common self-reported symptoms at the onset of illness were fever (93.5%), cough (81.1%), productive cough (41.3%), dyspnea (39.8%), and fatigue or myalgia (32.3%). They showed that dyspnea was significantly associated with the development of ARDS (p<0.001). However, in their study, none of the symptoms correlated significantly with the outcome [21]. Our analysis found that the most common patient comorbidities like hypertension and DM present in 22% and 21% of the patients, respectively. DM and COPD were significantly associated with ARDS at presentation (p=0.007 and p=0.03). DM, hypertension, and COPD were also significantly associated with mortality (p=0.02, p=0.01, and p=0.02, respectively). Bianca et al. also showed that DM, hypertension and especially cardiovascular disease are important risk factors for severity and mortality in COVID-19 infected people [23].

Our study revealed significantly aberrant coagulation parameters in admitted COVID-19 patients. We observed prolongations of both PT and APTT. PT was higher in non-survivors at D3 and D7. However, the result was not statistically significant (p=0.37, p=0.23 respectively). APTT was markedly prolonged among non-survivors at D0 and D7. The result was statistically significant (p=0.03, p=0.02). Langer et al. and Tang et al. observed minimum prolongation of APTT or PT in most patients [11,24]. D-Dimer was elevated in 38/68 (56%) patients at baseline. D-dimer levels were higher in non-survivors at D0, D3, D7 (p<0.001 on all occasions). Higher D-Dimer levels were also observed in patients with high SOFA scores at D0, D3, D7 (p<0.001, p=0.001, p=0.004, respectively). Similarly, in ARDS patients, the D-Dimer levels were increased compared to non-ARDS patients at D0, D3, D7 (p=0.001, p<0.001, p=0.002, respectively). Elevated D-dimer levels were associated with both in-hospital mortality and ARDS (figure 1 and figure 2). D-dimers are one of the fragments produced when plasmin cleaves fibrin to break down clots. The assays are routinely used as part of a diagnostic algorithm to exclude the diagnosis of thrombosis [25]. However, any pathologic or non-pathologic process that increases fibrin production or breakdown also increases plasma D-dimer levels. Examples include deep vein thrombosis/pulmonary embolism, arterial thrombosis, disseminated intravascular coagulation, and conditions such as pregnancy, inflammation, cancer, chronic liver diseases, post-trauma and surgery status, and vasculitis. Using the cut-off value of >2.13, we found that D-dimer levels upon admission for in-hospital mortality have an AUC of 0.86 (figure 1). Elevated D-dimer levels on admission (>2.13 mg/L) may identify patients at higher risk for in-hospital mortality and, therefore, may be informative about patients that may require intensive care and early intervention. Tang reported that the elevated D-dimer level in the in-patients with COVID-19 was related to higher mortality [11]. Yao et al. retrospectively analyzed D-dimer upon admission and identified a cut off value >2.14 mg/ml predicting in-hospital mortality with a sensitivity of 88.2% and specificity of 71.3% [26]. These findings implied that sustained hypercoagulable status and coagulation system activation are hallmarks of COVID-19 and provided strong evidence to support anticoagulation therapy in these patients. The natural anticoagulants [antithrombin, protein C and protein S] inhibit thrombosis and are essential for normal blood liquidity. Antithrombin is the major inhibitor of thrombin and factor Xa. To a lesser extent, an inhibitor of other serine proteases is generated during the coagulation process (factors IXa, XIa, and XIIa). The enzymatically active form of Protein C inhibits the clotting cascade at the levels of factors V and VIII, and Protein S serves as a cofactor in these reactions. In this study, the natural anticoagulants Protein C, Protein S, and Antithrombin levels decreased sequentially over time and in all categories. We did not observe consumption coagulopathy or disseminated intravascular coagulation (DIC) in our study. Our results align with similar studies that did not show a DIC state in COVID-19 patients [11]. We also measured plasma levels of tPA to know the balance between coagulation and fibrinolysis. We observed that tPA levels were higher in all COVID-19 patients. Impaired fibrinolysis has been suggested among COVID-19 patients, which may heighten thrombotic risk. tPA levels were higher in patients with high SOFA score at D0, D3, D7 (p=0.34, p=0.30, p=0.60 respectively). Patients with ARDS also had higher tPA levels D0, D3, D7 (p=0.53, p=0.90, p=0.40 respectively). Moreover, high tPA levels were also observed in non-survivors at D7 (p=0.12). The major source of these high levels of tPA among COVID-19 patients is likely endothelial cells. In addition to endothelial activation, direct infection and destruction of endothelial cells by SARS-CoV-2 may also potentiate the release of tPA. Nougier C. et al. in hospitalized COVID-19 patients also detected elevations of both PAI-1 and tPA, particularly among critically ill COVID-19 patients [27]. Therapies promoting fibrinolysis, such as administration of aerosolized or intravenous tPA, have been in trial in ARDS models with some promising preclinical results [28,29]. Pro-fibrinolytic therapy has been suggested as a potentially beneficial therapy in COVID-19 patients suffering from ARDS and is currently being tested in multiple clinical trials [30].

Limitations

More studies like this with a larger sample size may be undertaken to determine the effects of coagulation and fibrinolytic parameters in COVID-19 patients.

## Conclusions

To conclude, we comprehensively evaluated the clinical characteristics and coagulation parameters of all confirmed COVID-19 patients. We found that the incidence of ARDS was 34%, with a mortality of 13%. Comorbidities such as DM, hypertension and COPD were associated with poor outcomes (ARDS and mortality). We found that conventional clotting tests (PT, APTT, fibrinogen) were deranged in these patients. We also demonstrated that D-dimer levels should be monitored in COVID patients early after admission due to their association with outcome and mortality. We observed that the levels of natural anticoagulants fell during the illness, making them prone to coagulopathies. However, none were seen in this study. Elevated tPA levels were also found in our patients; fibrinolytic therapy may benefit COVID-19 patients suffering from ARDS.
